# Biomechanical Gender Differences in the Uninjured Extremity After Anterior Cruciate Ligament Reconstruction in Adolescent Athletes: A Retrospective Motion Analysis Study

**DOI:** 10.7759/cureus.35596

**Published:** 2023-02-28

**Authors:** Neeraj Vij, Sailesh Tummala, Jeffrey Vaughn, Anikar Chhabra, Hadi Salehi, Jenni Winters, Amber Browne, Kaycee Glattke, Joseph C Brinkman, Heather Menzer

**Affiliations:** 1 Department of Orthopedic Surgery, University of Arizona College of Medicine - Phoenix, Phoenix, USA; 2 Department of Orthopedic Surgery, Mayo Clinic, Phoenix, USA; 3 Department of Orthopedic Surgery, Phoenix Children's Hospital, Phoenix, USA

**Keywords:** gender specificity, female athlete, femoral adduction, sports medicine, drop vertical test

## Abstract

Introduction

Subsequent anterior cruciate ligament (ACL) injury is more common in the pediatric population and encompasses graft failure and subsequent contralateral tears. Females are at a higher risk. The purpose of the present study was to compare the knee valgus angles at initial contact, knee extension moments, anterior and lateral knee joint forces, hip flexion angles, hip adduction moments, and ankle inversion during the drop vertical test in the uninjured extremity between adolescent males and females who had previously undergone an anterior cruciate ligament reconstruction (ACLR).

Methods

This IRB-approved retrospective chart review included patients aged 8-18 years who were seen at the five to seven month postoperatively following ACL reconstruction. A total of 168 patients met our inclusion criteria (86 girls and 82 boys.) Using three-dimensional motion capture technology (CORTEX software, Motion Analysis Corp., Rohnert Park, CA), data were collected while the subject performed the drop vertical test over floor-mounted force plates (FP-Stairs, AMTI, Watertown, MA) under the direct supervision of a pediatric physical therapist. The Wilcoxon rank sum was used, and p < 0.05 was considered statistically significant.

Results

Females demonstrated a larger average knee joint extension moment (0.31 vs 0.28 N*m/kg, p = 0.0408), a larger anterior knee joint force at initial contact (3.51 vs. 2.79, N/kg, p = 0.0458), larger average hip flexion angle (41.50° vs. 35.99°, p = 0.0005), a smaller maximum hip adduction moment (0.92 vs. 1.16, N*m/kg, p = 0.0497), and a smaller average ankle inversion angle (5.08° vs. 6.41°, p = 0.03231). No significant differences were found regarding knee abduction angle or lateral knee joint force.

Conclusions

The biomechanical profile of the contralateral extremity varies significantly between the genders after ACLR. In the uninjured extremity, females may have larger hip flexion angles, smaller hip adduction moments, larger anterior knee joint forces, larger knee extension moments, and smaller ankle inversion angles as compared to males after ACLR. These findings may explain the higher incidence of subsequent contralateral injury in female adolescent athletes. Further work is required to develop a composite score that determines at-risk athletes.

## Introduction

Anterior cruciate ligament (ACL) injuries are common, with an incidence rate of up to five cases per 100,000 people [1]. Pediatric ACL injuries are more common, with an estimated incidence of 240 injuries per 100,000 [2]. Subsequent ACL reinjury is also more common in children. Subsequent ACL injury encompasses both graft failure and subsequent contralateral tear, with an overall incidence of 17.4% in the pediatric population [2]. Subsequent contralateral ACL tears are a devastating injury, with an incidence of 11.8% [3]. A failure to reproduce the native biomechanics of the entire lower extremity after anterior cruciate ligament reconstruction (ACLR) may expose the contralateral limb to an abnormal biomechanical environment during sporting activities. Many risk factors for subsequent contralateral tear have been identified. These include age less than 20 years [4-6], female gender [5,7], tobacco use [5], depression [5], and early return to sport [4]. A recent systematic review demonstrated that the rate of subsequent contralateral tears is 22.5% in females as compared to 8.7% in males [8].

Biomechanical risk factors have been shown to play an important role in first-time ACL injury risk [9]. Established risk factors for first-time tear risk include knee valgus angle, knee abduction moment, and knee external rotation moment [9]. The sex-based differences in the biomechanics of the native knee that lead to a female predisposition for first-time ACL injury have also been well described. Bates et al. demonstrated that females have lower anterior and lateral peak joint forces, lower external joint torque, and lower peak lateral joint forces through a cadaveric study (12 cadaveric knees) during robotically stimulated athletic tests [10]. Since the publication of this article, a handful of additional articles have explored gender-based kinematic and kinetic variations in the native adolescent knee [11-13].

The biomechanics leading to the risk of subsequent contralateral injury are less well-known. Paterno et al. demonstrated that knee valgus angles, knee extensor moments at initial contact, and hip internal rotation moment predicted the risk of subsequent ACL injury (including both graft failure and subsequent contralateral injury) [14]; however, it remains unclear whether these variables can be used to predict risk specifically of subsequent contralateral injury. Furthermore, the sex-based differences of the uninjured knee after contralateral ACL reconstruction remain unknown.

The purpose of the present study was to use the drop vertical test to compare the lower extremity biomechanics of the uninjured knee between adolescent males and females who had previously undergone an ACL reconstruction. The primary outcomes for this study were the knee valgus angles at initial contact, knee extension moments, and the anterior and lateral knee joint forces. The secondary outcomes for this study were hip flexion angles, hip adduction moments, and ankle inversion angles. We hypothesized that females would have larger knee valgus angles at initial contact [11], larger knee extension moments [10], larger anterior and lateral knee joint forces [10], larger hip flexion angles [13], larger hip adduction moments [12], and decreased ankle inversion [11].

## Materials and methods

General

This study was reviewed and approved by our institutional review board (IRB No. 19-376). The inclusion criteria were five to seven months after ACL reconstruction and ages 8-18 years. This age range was included to encompass the spectrum of pediatric ACL surgery. The exclusion criteria were prior knee injury or prior knee surgery to either limb apart from the ACLR and a history of other lower extremity injury.

Motion capture system

**Figure 1:**
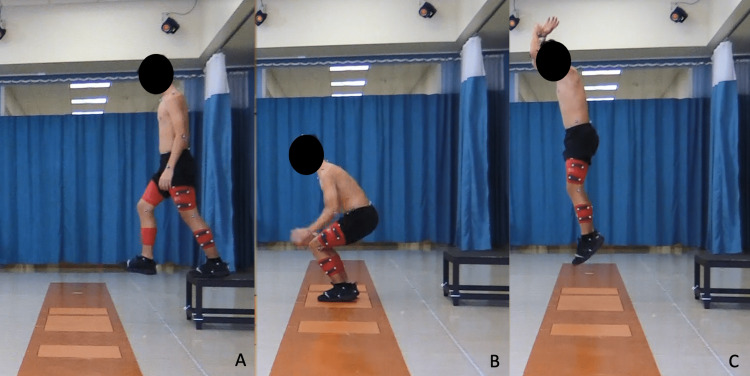
A depiction of a patient undergoing drop vertical testing before take-off (panel A), at landing (panel B), and at the peak of the jump (panel C).

The motion analysis was performed using a computerized video-based system (CORTEX software, Motion Analysis Corp., Rohnert Park, CA). Passive spherical markers with diameter of 9 mm were placed on the skin over bony landmarks per the Helen Hayes marker system. Motion data were collected at 120 Hz and force plate data at 1,080 Hz. Data were collected while the subject performed the drop vertical test over floor-mounted force plates (FP-Stairs, AMTI, Watertown, MA) under the direct supervision of a pediatric physical therapist (Figure 1). Three trials were conducted, and the trials were averaged per patient during processing.

Data collection

**Figure 2:**
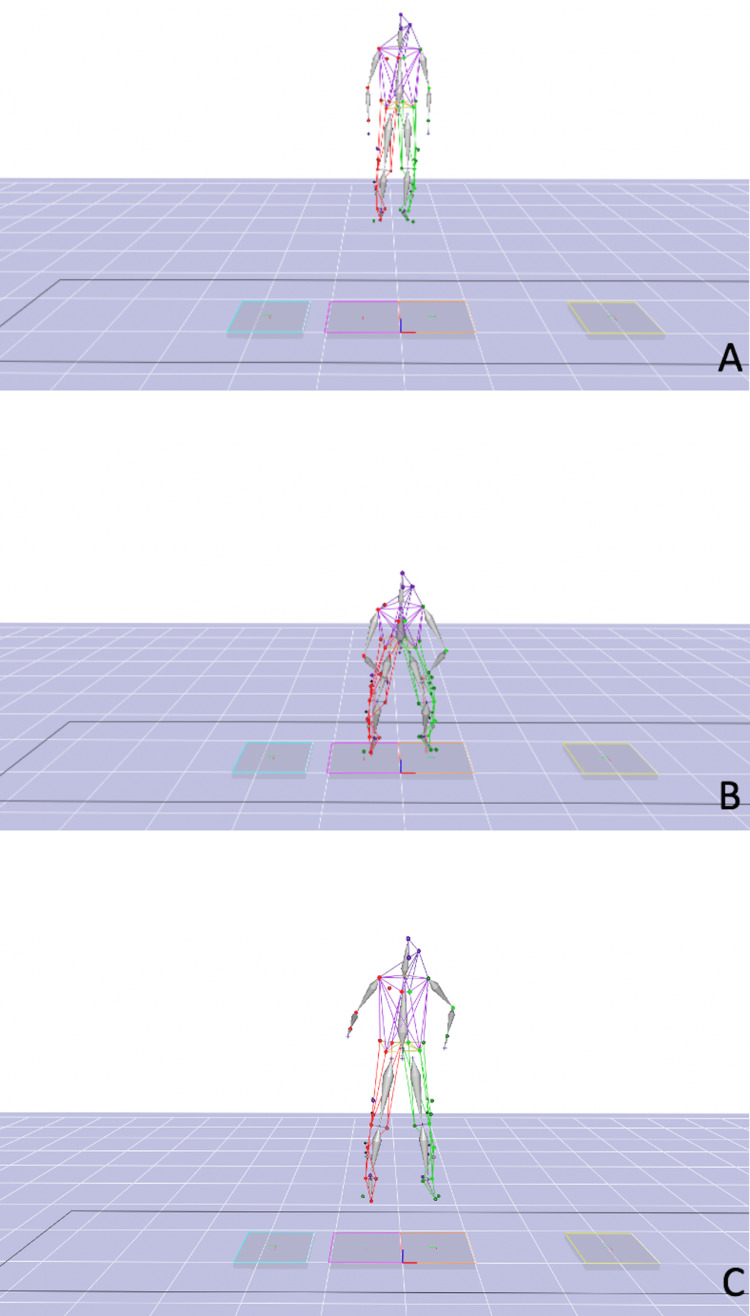
The simulated drop vertical test as measured by our motional analysis software.

A review of the patient's charts was performed to obtain the age (years), height (meters), weight (kilograms), and sex. The data elements collected for the motion analysis studies (Figure 2) were subdivided into kinematics, kinetics, and joint forces. The kinematic variables collected included the following: average hip flexion angle (°), maximum hip flexion angle (°), hip flexion angle at initial contact (°), knee valgus angle at initial contact (°), average ankle inversion angle (°), maximum ankle inversion angle (°), and ankle inversion angle at initial contact (°). The kinetic variables collected included: Average hip adduction joint moment (N*m/kg), maximum hip adduction joint moment (N*m/kg), average knee extension joint moment (N*m/kg), and maximum knee extension joint moment (N*m/kg). The joint force data collected included the following: average anterior knee joint force (N/kg), anterior knee joint force at initial contact (N/kg), lateral knee joint force (N/kg), and lateral knee joint force at initial contact (N/kg).

Confidentiality of data

This study was conducted in full accordance with all applicable hospital research policies and procedures and all applicable federal and state laws and regulations including 45 CFR 46 and the HIPAA Privacy Rule. Data were stored in a protected Excel file on a secure drive, using the most updated version of Excel (product version 16.0.13127.21668) in concordance with hospital policy. Only those individuals involved in the study had access to the data sheet. All patient-protected information was removed before statistical analysis.

Data abstraction and statistical analysis

Continuous variables were reported as means and standard deviations, and categorical variables were reported as frequencies and proportions. The Wilcoxon rank sum was used to compare continuous variables. P-values were two-sided, and p < 0.05 was considered statistically significant. Statistical analyses were performed using SAS 9.4 (SAS Institute, Cary, NC) and R Studio Version 1.0.153.

## Results

General

A total of 168 adolescent patients were included in the study; 82 patients were male and 86 patients were female. The male and female patients demonstrated similar characteristics (Tables 1, 2).

**Table 1:** Patient characteristics of the 81 males within our study population.

Parameter	Average Value	Median	Range	Standard Deviation
Age (years)	15.9	16	11–18	2.2
Height (cm)	188.8	172.8	123.2–191.6	11.4
Weight (kg)	74.2	69.3	31.2–174.4	21.6
BMI (m/kg^2^)	25.0	23.3	12.4–47.5	5.8

**Table 2:** Patient characteristics of the 87 females within our study population.

Parameter	Average Value	Median	Range	Standard Deviation
Age	15.5	15	10–18	1.69
Height	166.4	166	141.1–191.3	9.47
Weight	69.13	65.2	41.5–125.4	16.37
BMI	24.87	23.68	17.2–40.25	5.08

Our results are summarized in Table 3 and in the following sections.

**Table 3:** A summary of the results of our study.

	Parameter Name (Units)	Female			Male			P-Value	Total		
		Mean (SD)	Median	Q1, Q3	Mean (SD)	Median	Q1, Q3		Mean (SD)	Median	Q1, Q3
Kinematics	Average hip flexion angle (°)	41.50 (4.53)	42.55	35.23, 48.48	35.99 (5.24)	36.58	30.46, 40.78	0.00051	38.81 (4.77)	39.45	32.24, 46.60
	Maximum hip flexion angle (°)	99.50 (5.85)	100.19	92.94, 106.55	98.58 (7.83)	98.94	92.82, 107.82	0.71161	99.04 (6.95)	99.67	92.91, 107.28
	Hip flexion angle at initial contact (°)	47.64 (3.28)	47.8	40.76, 55.73	45.79 (4.71)	46.12	37.58, 53.30	0.27641	46.73 (4.37)	46.53	39.60, 54.85
	Knee valgus angle at initial contact (°)	4.78 (2.56)	4.83	1.66, 7.95	4.76 (2.96)	3.91	1.83, 7.22	0.67531	4.77 (2.74)	4.45	1.81, 7.53
	Average ankle inversion angle (°)	5.08 (1.45)	3.81	1.17, 8.73	6.41 (1.28)	6.63	2.66, 9.26	0.03231	5.73 (1.39)	4.89	1.81, 9.04
	Maximum ankle inversion angle (°)	15.60 (3.46)	14.76	10.74, 18.70	16.62 (3.95)	16.46	13.05, 20.30	0.03641	16.10 (3.79)	15.78	11.61, 19.29
	Ankle inversion angle at initial contact (°)	8.63 (3.55)	8.05	4.89, 13.30	10.06 (2.58)	10.53	6.25, 13.77	0.10591	9.33 (2.85)	9.17	5.25, 13.54
Kinetics	Average hip adduction joint moment (N*m/kg)	0.06 (0.04)	0.06	0.03, 0.09	0.06 (0.03)	0.06	0.02, 0.09	0.74621	0.06 (0.04)	0.06	0.02, 0.09
	Maximum hip adduction joint moment (N*m/kg)	0.92 (0.26)	0.8	0.48, 1.3	1.16 (0.19)	0.99	0.61, 1.41	0.04971	1.04 (0.24)	0.91	0.55, 1.32
	Average knee extension joint moment (N*m/kg)	0.31 (0.08)	0.32	0.23, 0.41	0.28 (0.13)	0.26	0.19, 0.36	0.04081	0.29 (0.07)	0.29	0.20, 0.38
	Maximum knee extension joint moment (N*m/kg)	2.56 (0.90)	2.36	2.00, 2.81	2.36 (0.79)	2.35	1.92, 2.65	0.20041	0.86 (0.83)	0.88	0.19, 1.42
Joint forces	Average anterior knee joint force (N/kg)	1.27 (0.49)	1.33	0.98, 1.65	1.17 (0.54)	1.13	0.85, 1.45	0.05231	1.22 (0.51)	1.25	0.90, 1.55
	Anterior knee joint force at initial contact (N/kg)	3.51 (1.65)	3.72	1.92, 5.45	2.79 (1.22)	2.65	0.54, 4.40	0.04581	3.16 (1.45)	3.14	1.38, 5.07
	Average lateral knee joint force (N/kg)	0.96 (0.65)	0.83	0.40, 1.41	1.14 (0.77)	0.85	0.50, 1.28	0.52881	1.05 (0.71)	0.85	0.44, 1.31
	Lateral knee joint force at initial contact (N/kg)	1.89 (0.93)	1.78	1.29, 2.29	2.27 (0.77)	1.88	1.53, 2.57	0.24741	2.07 (0.91)	1.81	1.44, 2.45

Hip

**Figure 3:**
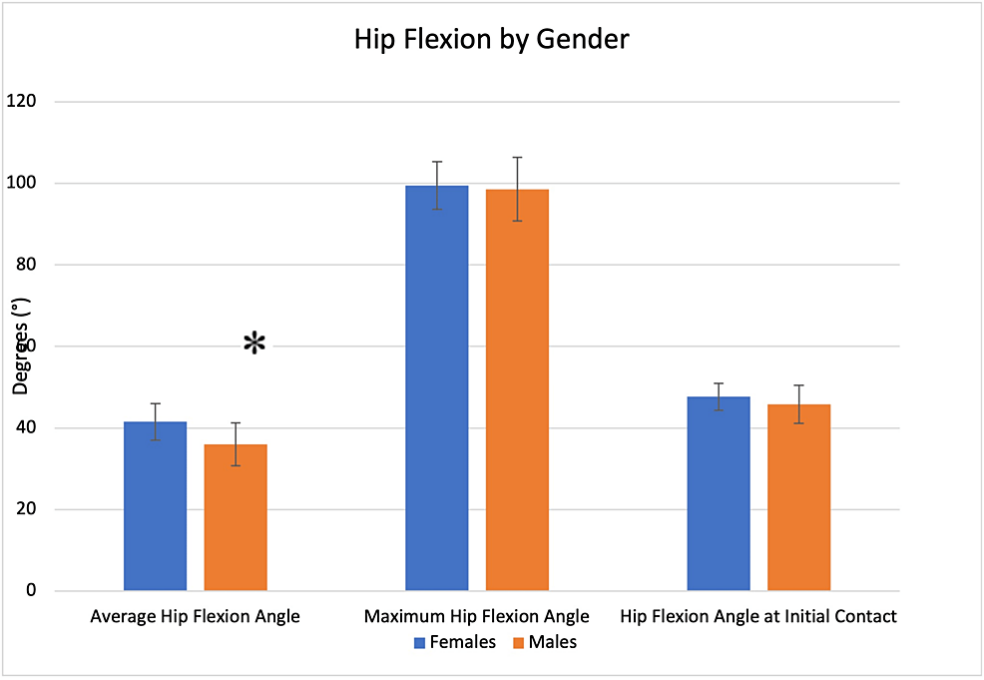
Hip flexion profile by gender. Significant findings are denoted with an asterisk (*).

Females demonstrated a larger average hip flexion angle (41.50° vs. 35.99°, p = 0.0005). No significant differences were seen in maximum hip flexion angle or hip flexion angle at initial contact (Figure 3).

**Figure 4:**
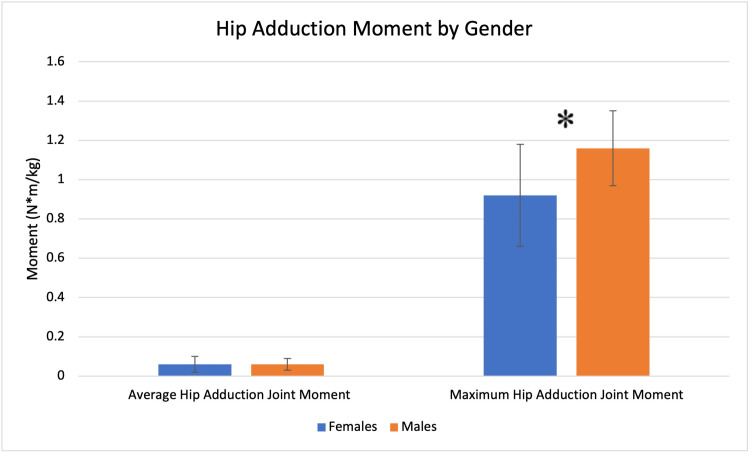
Hip adduction moment profile by gender. Significant findings are denoted with an asterisk (*).

Females demonstrated a smaller maximum hip adduction moment (0.92 vs. 1.16, N*m/kg, p = 0.0497), but no significant differences were seen in the average hip adduction moment (Figure 4).

Knee

**Figure 5:**
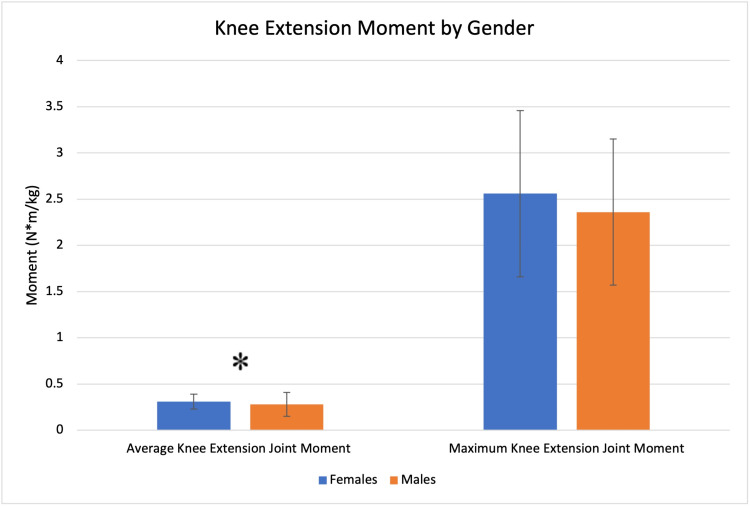
Knee extension moment profile by gender. Significant findings are denoted with an asterisk (*).

There were no statistically significant differences in knee valgus angle at initial contact. Females demonstrated a larger average knee joint extension moment (0.31 vs 0.28 N*m/kg, p = 0.0408, Figure 5).

**Figure 6:**
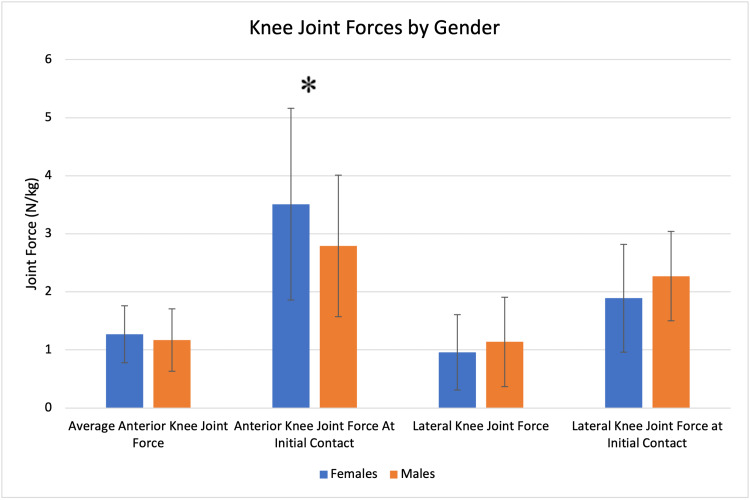
Knee joint force profile by gender. Significant findings are denoted with an asterisk (*).

Females also demonstrated a larger anterior knee joint force at initial contact (3.51 vs. 2.79, N/kg, p = 0.0458), but no significant differences were seen in the average anterior knee joint force or lateral knee joint forces (Figure 6).

Ankle

**Figure 7:**
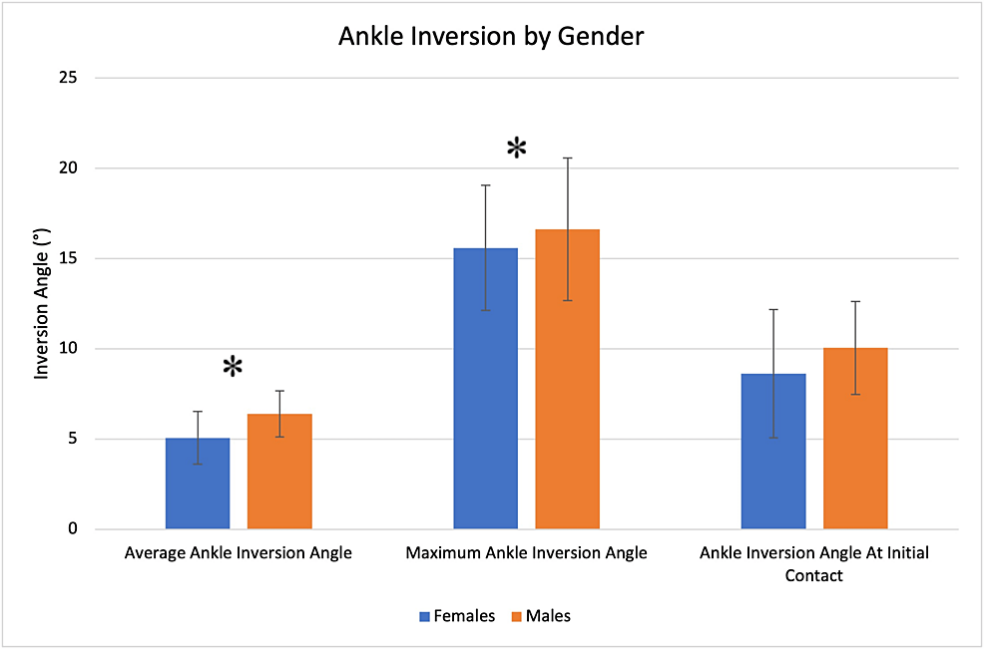
Ankle inversion profile by gender. Significant findings are denoted with an asterisk (*).

Females demonstrated a smaller average ankle inversion angle (5.08° vs. 6.41°, p = .03231) and a smaller maximum ankle inversion angle (15.60° vs. 16.62°, p = 0.0346). No significant difference was seen in the ankle inversion angle at initial contact (Figure 7).

## Discussion

The purpose of this retrospective chart review was to compare the lower extremity profile of the uninjured limb between males and females who had previously undergone an ACL reconstruction. We hypothesized that females would have larger knee valgus angles at initial contact [11], larger knee extension moments [10], larger anterior and lateral knee joint forces [10], larger hip flexion angles [13], larger hip adduction moments [12], and decreased ankle inversion [11]. In our study, females demonstrated a larger average knee joint extension moment (0.31 vs 0.28 N*m/kg, p = 0.0408), a larger anterior knee joint force at initial contact (3.51 vs. 2.79, N/kg, p = 0.0458), larger average hip flexion angle (41.50° vs. 35.99°, p = 0.0005), a smaller maximum hip adduction moment (0.92 vs. 1.16, N*m/kg, p = 0.0497), and a smaller average ankle inversion angle (5.08° vs. 6.41°, p = 0.03231). No significant differences were found regarding knee abduction angle or lateral knee joint force.

Hewett et al. were one of the first to explore the extremity biomechanics after ACLR topic and demonstrated that knee abduction angle, knee abduction moment, and hip adduction moments were associated with first-time anterior cruciate ligament injury in females [9]. In our study, females after ACLR did demonstrate smaller hip adduction moments in the uninjured limb. This may explain the higher incidence of subsequent contralateral ACL injuries in females who have previously undergone ACLR [5,7].

The literature surrounding the conversation of biomechanical risk factors for subsequent ACL tear is more sparse. Paterno et al. explored the role of a prospective biomechanical screening protocol [2]. Following the motion analysis assessment, patients who had undergone ACLR and had returned to their prior level of sports were followed for a period of 12 months. In their study, there were a total of 10 subsequent contralateral tears and only three graft failures consistent with prior literature [3]. They also demonstrated knee valgus and knee extensor moments are predictors of subsequent ACL injury. Our study found that knee extension moments of the uninjured limb in patients after ACLR were significantly greater in female patients as compared to males. These findings may suggest that knee extensor moments may be part of the underlying biomechanical predisposition for the higher subsequent ACL injury rate seen in females. 

A handful of studies have found differences in ankle kinematics between the genders in the native or uninjured extremity [11,15,16]. Ford et al. demonstrated that female athletes have lower ankle eversion maximums and larger ankle inversion maximums as compared to males on both the dominant and non-dominant leg [11]. These findings correlate well with the ankle inversion differences seen in our study in the contralateral limb of patients who had previously undergone ACLR. It is interesting to note that in both our study and Ford et al.’s study, the significance was not achieved when testing inversion at initial contact. However, there is little consensus regarding the role of ankle inversion in the risk of first-time or subsequent ACL injury. Further studies may reveal the predictive value of this variable.

Of note to this discussion is the role that anticipation may play in the ability of the nervous system to protect the human body. A few articles in the literature studied the kinematics and kinetics of the knee in the context of unanticipated cutting maneuvers. These articles found gender differences with regard to knee abduction [11] and hip and knee flexion [15]. The lack of irreproducibility of the abduction kinematics in our study may be explained by the testing choice. Though the drop vertical test is an important dynamic test that tests the functional resistance to collapsing into valgus, it may not best represent the in-game environment [11,15].

As a retrospective biomechanical study with the absence of clinical data, this study is unable to make any inferences regarding the causality of any of the identified parameters with regard to risk of subsequent contralateral injury after ACLR. Our study encompasses athletes from many disciplines, and thus we are unable to draw any inferences regarding the sport specificity of any of the identified biomechanical parameters. Lastly, our study includes individuals ranging from highly competitive athletes to recreational sports participants. Thus, it is unclear whether these findings can be extrapolated to high-level adolescent athletes.

## Conclusions

The biomechanical profile of the contralateral extremity varies significantly between the genders after ACLR. Females may have larger hip flexion angles, smaller hip adduction moments, larger anterior knee joint forces, larger knee extension moments, and smaller ankle inversion angles as compared to males in the uninjured knee after ACLR. These findings may explain the higher incidence of subsequent contralateral injury in female adolescent athletes. Further work is required to develop a composite score that can identify at-risk athletes.
